# CdSe Quantum Dot (QD)-Induced Morphological and Functional Impairments to Liver in Mice

**DOI:** 10.1371/journal.pone.0024406

**Published:** 2011-09-29

**Authors:** Wei Liu, Shuping Zhang, Lixin Wang, Chen Qu, Changwen Zhang, Lei Hong, Lin Yuan, Zehao Huang, Zhe Wang, Sijin Liu, Guibin Jiang

**Affiliations:** 1 State Key Laboratory of Environmental Chemistry and Ecotoxicology, Research Center for Eco-Environmental Sciences, Chinese Academy of Sciences, Beijing, China; 2 Department of Microbiology, Comprehensive Cancer Center, University of Pennsylvania Medical School, Philadelphia, Pennsylvania, United States of America; East Carolina University, United States of America

## Abstract

Quantum dots (QDs), as unique nanoparticle probes, have been used in *in vivo* fluorescence imaging such as cancers. Due to the novel characteristics in fluorescence, QDs represent a family of promising substances to be used in experimental and clinical imaging. Thus far, the toxicity and harmful health effects from exposure (including environmental exposure) to QDs are not recognized, but are largely concerned by the public. To assess the biological effects of QDs, we established a mouse model of acute and chronic exposure to QDs. Results from the present study suggested that QD particles could readily spread into various organs, and liver was the major organ for QD accumulation in mice from both the acute and chronic exposure. QDs caused significant impairments to livers from mice with both acute and chronic QD exposure as reflected by morphological alternation to the hepatic lobules and increased oxidative stress. Moreover, QDs remarkably induced the production of intracellular reactive oxygen species (ROS) along with cytotoxicity, as characterized by a significant increase of the malondialdehyde (MDA) level within hepatocytes. However, the increase of the MDA level in response to QD treatment could be partially blunted by the pre-treatment of cells with beta-mercaptoethanol (β-ME). These data suggested ROS played a crucial role in causing oxidative stress-associated cellular damage from QD exposure; nevertheless other unidentified mediators might also be involved in QD-mediated cellular impairments. Importantly, we demonstrated that the hepatoxicity caused by QDs *in vivo* and *in vitro* was much greater than that induced by cadmium ions at a similar or even a higher dose. Taken together, the mechanism underlying QD-mediated biological influences might derive from the toxicity of QD particles themselves, and from free cadmium ions liberated from QDs as well.

## Introduction

With large production and wide use of nanomaterials, the odds of human exposure to them increases significantly under occupational, medical and environmental settings, and adverse biological influences and potential risks on human health are publicly concerned [1], [2], [3], [4]. Although the pernicious effects of nanomaterials have been documented from a number of publications, the understanding of their health and safety characteristics currently lags far behind their own development [5]. Quantum dots (QDs) represent a family of fluorescent semiconductor nanoparticles with high annual yield [6]. Due to the distinctive characteristics (e.g. narrow emission peak and constant excitation profile), QDs are potent substances used in imaging [7]. And QDs have been applied in *in vivo* fluorescence imaging as nanoparticle probes for lymph nodes and cancers [8], [9], [10]. Meanwhile, the toxicity and health risk from exposure (including potential exposure from environment and daily life) to QDs are largely concerned.

Numerous studies have documented a variety of toxicities of QDs to cells [11]. However, to date, most toxicological assessments were performed in cell culture-based assay systems, and the current data from limited animal studies keep inconclusive. Additionally, the *in vivo* toxicity differentiated in various studies due to variations in particle size, dose, surface modification and exposure conditions [12]. Although the accumulation of QDs in organs was closely investigated, the consequent biological effects on organs were rarely addressed upon QD exposure and accumulation. Moreover, little is known about the molecular mechanisms responsible for QD-mediated biological events and cytotoxicity.

To elucidate whether there is damage to organs with preferential accumulation of QDs in them, we performed both acute and chronic exposure of CdSe QDs in adult mice. Data from the current study suggest that liver is the predominant site for QD accumulation, which leads to significant hepatoxicity as reflected by morphological alternation to the hepatic lobules and increased antioxidant capability of hepatocytes. Importantly, we demonstrated that QD-stimulated oxidative stress is the major mediator of cytotoxicity based on the results from the animal work and *in vitro* cultured cells. The present study suggests ROS plays a crucial role in causing oxidative stress-associated cellular damage from QD exposure, and other unidentified mediators derived from QDs might also be involved.

## Results and Discussion

**Figure 1:**
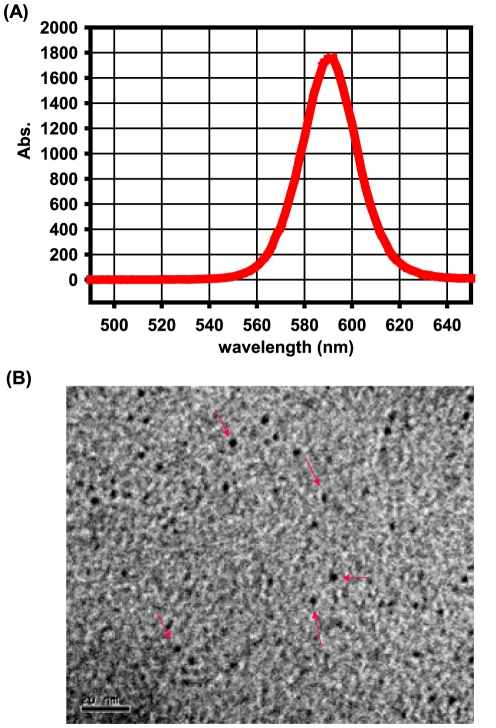
The TEM image and the fluorescent curve of QDs used in the present study. (A) The evaluation of the fluorescence spectrum of the QDs. (B) A representative TEM image showed the shape and morphology of the QDs. Arrows indicated the QD particles. The scale was 20 nm, and the original magnification was 500,000×.

The TEM assessment showed the shape and morphology of the QDs used in this study (Fig. 1A). The analysis based on the nanoparticle size analyzer suggested that the diameter of QDs was about 4 nm. And the evaluation of the fluorescence spectrum indicated that the maximum emission wavelength was 590±10 nm, and maximum half width was ≤32 nm (Fig. 1B).

**Figure 2:**
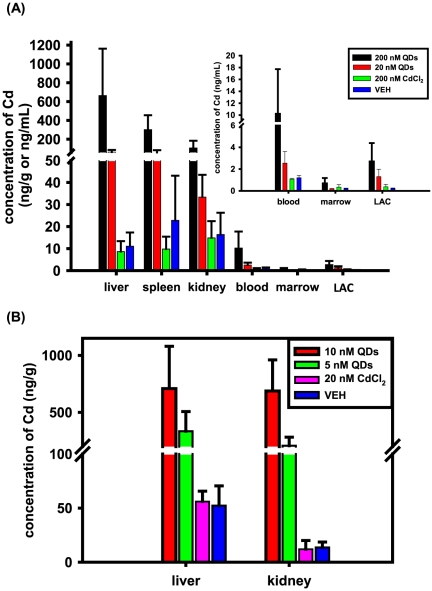
The distribution of QDs in various tissues from mice with acute exposure (A) and chronic exposure (B). QD content reflected by the cadmium amount was shown in livers, spleens, kidneys, blood, marrow and lavages from abdominal cavities (LAC). The graph for the QD content in blood, marrow and LAC was enlarged and presented in the upper-right corner of the (A). Result were presented as mean±SD (n = 8–10).

To evaluate the biological influences of QD accumulation in tissues from mice with the acute and chronic exposure to QDs, we first assessed QD content in various tissues. Regarding the results from the acute exposure (Fig. 1A), QDs reflected by the cadmium amount were predominantly spread into livers, spleens and kidneys, especially livers, from mice with exposure to CdSe QDs for both doses at 20 nM and 200 nM, consistent with previous studies [13], [14], [15]. The cadmium concentration in above tissues from the 200 nM QD-treated mice was significantly higher than that from the 20 nM QD-treated mice (P = 0.017 for liver, P<0.001 for kidney and P = 0.04 for spleen, Fig. 1). In contrast, only a little cadmium was found in blood and bone marrow, and importantly, little cadmium was detected in the lavages from the abdominal cavities, where CdSe solution was administered (Fig. 1). This finding suggested CdSe particles were quickly taken away and distributed into tissues within 48 hrs post the intraperitoneal administration. To better understand the pattern of tissue distribution and accumulation of QD particles in mice, we also performed a chronic exposure at lower concentrations, 5 nM and 10 nM QDs and 20 nM CdCl_2_ for 6 wks. The pattern of QD accumulation in tissues was similar to that from the acute exposure as described above, and the cadmium concentration in liver from the 20 nM QD-treated mice for 6 wks was comparable to that from the 200 nM QD-treated mice for 48 hrs (Fig. 2B). Importantly, kidney tended to retain more QD particles over 6 wks under the chronic treatment, as the cadmium concentration in kidney from the 20 nM QD-treated mice for 6 wks was similar to that in liver, and was much higher than that from the 200 nM QD-treated mice for 48 hrs (Fig. 2B).

**Figure 3:**
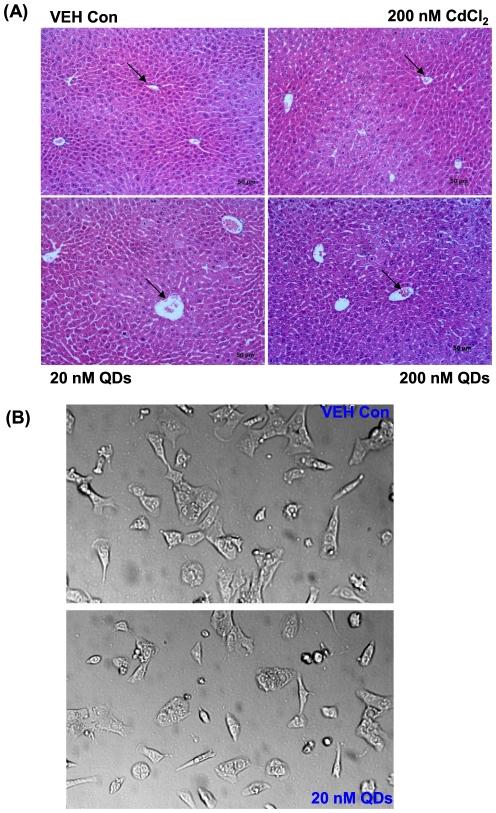
The hepatoxicity from QDs. (A) The toxicity to liver tissues from mice upon QD treatment evaluated by histological examination. Arrows indicate central veins in the core of hepatic cords. The original magnification was 200×, and the scale was 50 µm. (B) The cytotoxicity of QDs to *in vitro* cultured mouse hepatocytes, Hepa 1–6 cells. The phase-contrast images showed the cell morphologies in the 20 nM QD group and the vehicle control group. The original magnification was 200×.

Substantial QD acquisition in livers, spleens and kidneys might result in injuries to these organs. We therefore evaluated tissue changes from the acute and chronic exposure at the microscopic level via histological examination. No noticeable alternation was detected in the spleens and kidneys based on histological examination for both the acute and chronic exposure (data not shown). In contrast, there were dramatic morphological alternations to the hepatic lobules in livers from mice treated with 20 nM or 200 nM QDs for 48 hrs, as indicated by disordered hepatic cords and enlarged central veins (Fig. 3A), compared to those from the vehicle control mice. Hepatic impairments observed in livers from mice treated with 5 nM and 10 nM QDs for 6 wks were similar to those in livers from mice treated with 20 nM and 200 nM for 48 hrs (data not shown). No injury was detected in the livers of mice treated with 200 nM CdCl_2_ for 48 hrs (Fig. 3A), and in the livers of mice treated with 20 nM CdCl_2_ for 6 wks (data not shown), compared to the vehicle control.

**Figure 4:**
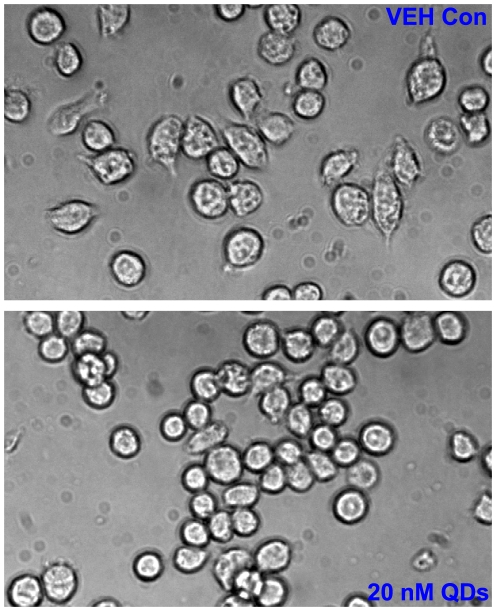
The cytotoxicity of QDs to *in vitro* cultured murine monocyte-macrophage J774A.1 cells. The phase-contrast images showed the alternations to cell morphology upon 20 nM QD treatment, compared to the vehicle control. The original magnification was 200×.

To further study the cytotoxicity caused by QDs at the cellular level, we delineated morphological alternations upon QD exposure by employing two types of *in vitro* cultured cells, murine hepatoma cells, Hepa 1–6, and monocyte-macrophage, J774A.1 cells. For this purpose, Hepa 1–6 might be able to represent hepatocytes from liver, and J774A.1 could represent Kuppfer cells from liver. Hepa 1–6 and J774A.1 cells were treated with 20 nM CdCl_2_, 5 nM, 10 nM and 20 nM QDs for 24 hrs and 48 hrs, and then cellular morphologies were assessed under a phase-contrast microscope. As shown in Fig. 3B, Hepa 1–6 cells treated with 20 nM QDs for 24 hrs were condensed compared to the control cells, and the average size of the cells became smaller than that of the control cells. Similar morphological alternations were observed in cells treated with 20 nM QDs for 48 hrs (data not shown); however, no significant alternations to cellular morphologies were recognized in cells treated with 20 nM CdCl_2_, 5 nM and 10 nM QDs for 24 hrs and 48 hrs (data not shown). As shown by phase-contrast microscopy, the control J774A.1 cells formed typical macrophages with outward protrusions at the peripheral membrane after 24 hr culture (Fig. 4). In contrast, J774A.1 cells upon 20 nM QD exposure were round and condensed with fewer outward protrusions, suggesting macrophagic differentiation was impaired by QDs (Fig. 4). Similar observations to J774A.1 cells were recognized in response to 20 nM QDs for 48 hrs (data not shown). No significant morphological differences were noted in cells treated with 20 nM CdCl_2_, 5 nM and 10 nM QDs for 24 hrs and 48 hrs, compared to the control cells (data not shown). Importantly, FACS analysis of apoptosis did not suggest cell death at 24 and 48 hrs upon exposure to CdCl_2_ and QDs at various concentrations described above using FITC-Annexin V and PI stains (data not shown). These findings collectively demonstrated that QD particles robustly induced cytotoxicity to hepatocytes and macropahges, and potently attenuated cell differentiation without causing cell death, in parallel to the observations of the *in vivo* hepatoxicity (Fig. 3A).

**Figure 5:**
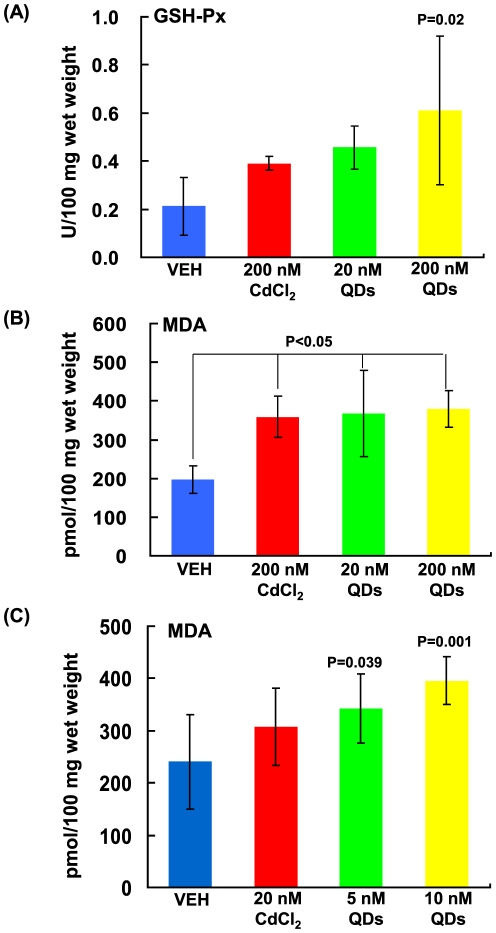
QD-induced oxidative stress *in vivo*. The levels of GSH-Px (A) and MDA (B) in livers of mice treated with 200 nM CdCl_2_, 20 nM and 200 nM CdSe QDs for 48 hrs. (C) The levels of MDA in livers of mice treated with 10 nM and 5 nM CdSe QDs and 20 nM CdCl_2_ for 6 wks. Results were presented as mean±SD (n = 8–10).

Oxidative stress is currently believed to be the main modulator of toxicity upon exposure to nanomaterials including both ultrafine sphere-like nanoparticles (e.g. QDs and nanosliver) and long fiber-like carbon nanotubes [1], [16], [17]. Thus, to shed light on the mechanism responsible for hepatic damage induced by QDs, we evaluated oxidative stress stimulated by QDs. The glutathione peroxidase (GSH-Px, as a reactive oxygen species scavenger) activity in the livers was assayed, as shown in Fig. 5A. There was an increase in the GSH-Px activity in the livers from mice treated with 20 nM and 200 nM QDs for 48 hrs, especially for 200 nM QDs (P = 0.02), compared to the vehicle control. Although not so robust as QDs, CdCl_2_ exposure also increased the hepatic GSH-Px activity (P>0.05, Fig. 5A), in agreement with a previous study [18]. The enhancement in the activity of the antioxidation indicated that the oxidative stress elevated the antioxidant capability of hepatocytes. Lipid peroxidation is considered as an important index for the identification of oxidative stress. Decomposition of lipid peroxides generates a lot of products including malondialdehyde (MDA). MDA is widely used as a marker of lipid peroxidase. We observed a significant increase of MDA content in the livers from the acute QD-treated mice compared to that in the control mice (P<0.05, Fig. 5B). The MDA level in the livers of acute CdCl_2_ –treated mice was also increased compared to that in the control mice (P<0.05, Fig. 5B), similar to the previous studies [19], [20]. Similarly in the chronic exposure, MDA content in the livers from the mice treated with 5 nM and 10 nM QDs (especially 10 nM QDs) for 6 wks was significantly increased compared to that in the control mice (P<0.05, Fig. 5C). The MDA level in the livers from mice exposed to 20 nM CdCl_2_ for 6 wks was also increased, but not statistically significant, compared to that in the control mice (P>0.05, Fig. 5C). It has been documented that cadmium causes hepatic oxidative stress in mice, thus leading to liver damage characterized by increased lipid peroxidation and altered antioxidant system [21], [22]. Therefore, oxidative stress stands for a major mechanism of acute and chronic cadmium toxicity. These data together suggested that the accumulation of QDs in livers resulted in hepatic injury mainly via oxidative stress, and the hepatic toxicity from QDs appeared greater than that from an equal amount of CdCl_2_. Thus, QDs might cause more severe hepatic damage than CdCl_2_ in that QDs could readily spread and deposit in liver in comparison to cadmium ions (as shown in Fig. 1). Moreover, owing to the superfine size, CdSe particles might have the capability to readily enter hepatocytes, where they could trigger severe intracellular impairments [23], [24], [25].

**Figure 6:**
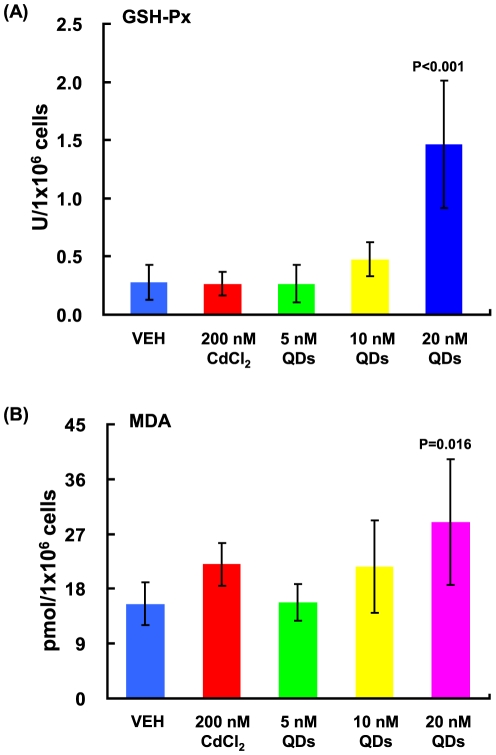
QD-induced oxidative stress *in vitro*. The levels of GSH-Px (A) and MDA (B) in the Hepa 1–6 cells with exposure to 200 nM CdCl_2_, 5 nM, 10 nM and 20 nM CdSe QDs for 48 hrs. Results were presented as mean±SD (n = 3–4).

To confirm the novel hepatic toxicity induced by QDs, we performed similar assays *in vitro* by treating murine hepatic Hepa 1–6 cells with CdCl_2_ and QDs. 24 hrs post treatment, increased GSH-Px activity was observed in QD-treated cells in a dose-dependent manner compared to the vehicle control (P<0.001, reflected by the one-way ANOVA test), particularly in the 20 nM QD-treated cells (P<0.001) (Fig. 6A). However, no significant increase in the GSH-Px activity was detected in the CdCl_2_-treated cells, even though the concentration of CdCl_2_ (200 nM) was 10 times higher than the highest concentration for QDs (20 nM) used in this study (Fig. 6A). This finding indicated that the intracellular antioxidant system was considerably stimulated by QD-triggered oxidative stress to cells, but not by CdCl_2_ treatment. Regarding the induction of MDA, QDs enhanced the MDA level in a dose-dependent manner compared to the vehicle control (P = 0.059, reflected by the one-way ANOVA test), particularly in the 20 nM QD-treated cells (P = 0.016) (Fig. 6B). CdCl_2_ at 200 nM also increased the MDA production compared to the vehicle control (P = 0.059); however, the increase was less than that in the 20 nM QD treatment (Fig. 6B).

**Figure 7:**
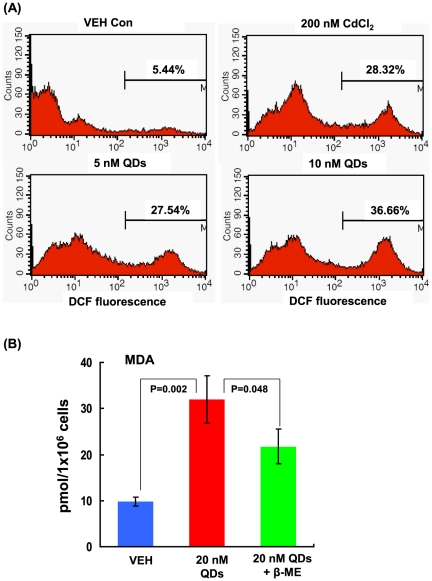
ROS and MDA generation in Hepa 1–6 cells upon QD treatment. (A) DCF fluorescence in cells was measured by FACS analysis after 6-hr treatment with 200 nM CdCl_2_, 5 nM, and 10 nM CdSe QDs. (B) The concentrations of MDA in Hepa 1–6 cells upon 20 nM CdSe QDs for 6 hrs with or without β-ME. Results were presented as mean±SD (n = 3).

To illustrate the mechanism by which QDs promoted oxidative stress *in vivo* and *in vitro*, we investigated intracellular reactive oxygen species (ROS) production in response to QDs. As presented in Fig. 7A, QDs largely induced the production of intracellular ROS in Hepa 1–6 cells at a low concentration of 5 nM for 6 hrs, similar to that upon 200 nM CdCl_2_ (27.54% VS 28.32%). At 10 nM, QDs generated more ROS compared to 5 nM (36.66% VS 27.54%) (Fig. 7A). To demonstrate that ROS played a critical role in mediating oxidative stress upon QD exposure, we pre-treated cells with a potent ROS scavenger, beta-mercaptoethanol (β-ME) [26], [27], to quench intracellular ROS. As a result, the cytotoxicity to Hepa 1–6 cells characterized by the intracellular MDA level was dramatically reduced compared to that in cells treated with 20 nM QDs only (P<0.05, Fig. 7B). However, the MDA level in cells upon 20 nM QD exposure with pre-treatment of β-ME was still higher than that in the control cells (P<0.05, Fig. 7B). These data together suggested ROS played a crucial role in mediating cytotoxicity caused by QDs; however, other unidentified mediators derived from QDs might also be contributive to the cellular impairments.

Taken together, CdSe particles could readily distribute into various organs upon the *in vivo* administration, and liver appeared to be the predominant site for the QD accumulation. QDs induced dramatic hepatic toxicity *in vivo* and *in vitro*, which was much greater than that induced by cadmium ions at a similar or even a higher dose. The mechanism responsible for QD-triggered hepatoxicity might derive from the toxicity from QD particles themselves and cadmium-stimulated oxidative stress as well. The toxic effect of QDs might be partially due to the liberation of cadmium ions from the QD core [28], [29], [30], [31], and the toxicity of free cadmium ions (such as cadmium-stimulated ROS) is presumably an important contribution to the overall toxicity of QDs [6], [7]. Additionally, QDs *per se* as fine nanoparticles represent distinct toxic characteristics from cadmium, such as size/shape-dependent effects, and aggregation- and surface composition-associated influences. The active QD cores are involved in free radical formation (such as ROS), and free radical-mediated oxidative stress is considered as another crucial contribution to the QD toxicity [32], [33], [34]. To sum up, the influences from both cadmium and QD cores together build up novel QD toxicities, including the hepatoxicity as discussed in the current study.

## Materials and Methods

### Chemicals and reagents

CdSe QDs without any modification and functional coating were obtained from Wuhan Jiayuan Quantum Dots Co., LTD., China. The QDs were stored in dark at 4°C. The nanomaterials were characterized prior to experiments. The morphologies of QDs were assessed by transmission electron microscopy (TEM, Hitachi H-7500, Japan) as previously reported [35]. The particle sizes were assayed using a nanoparticle size analyzer (N5, Backman, USA). The fluorescence spectrum was measured using a Hitachi F-4500 spectrofluorometer (Hitachi Co. Ltd., Tokyo, Japan). Prior to administration in animals, QD particles were dissolved in borate buffer.

### Animal work

All mouse care and experimentation were approved by the Committee of Animal Care at the RCEES, Chinese Academy of Sciences. Regarding the acute exposure, six-week-old male Kunming mice were administrated intraperitoneally with 200 nM CdCl_2_ (292.8 ng/kg body weight), 20 nM (30.6 ng/kg body weight) and 200 nM (305.6 ng/kg body weight) CdSe QDs in 50 mM borate buffer. Control mice received borate buffer only. Mice were then sacrificed 48 hrs post injection, and blood, bone marrow, lavage from abdominal cavity, liver, spleen and kidney samples were collected. With respect to the chronic exposure, mice received 20 nM CdCl_2_ (29.3 ng/kg body weight), 5 nM (7.6 ng/kg body weight) and 10 nM (15.3 ng/kg body weight) QDs for 6 wks, and mice were sacrificed at 24 hrs after the final injection. Blood and tissue samples were collected similar to the above-mentioned experimentation for the acute exposure. Histological examination was carried out with H&E staining as previously described [36].

### Determination of cadmium mass

The cadmium mass in blood, organs and lavages were measured using the ICP-MS method according to the protocol described in a previous study [14]. Briefly, samples were quantified by weight (organs) or volume (blood and lavages) and digested with strong oxidation-acid solution (a mix of nitric acid and hydrogen peroxide with a proportion of 3∶2) overnight. Then, the primarily digested samples were digested thoroughly at 180°C for 20 mins by microwave assisted digestion (MAD, Mars5 HP500, CEM Corporation, USA). Finally, concentrations of cadmium in these samples were quantified using inductively coupled plasma mass spectrometry (ICP-MS, Agilent 7500, USA).

### Cell culture

Mouse hepatoma Hepa 1–6 cells and monocyte-macrophage J774A.1 cells (both purchased from the Shanghai Cell Bank of Type Culture Collection of CAS) were cultured in 1640 and DMEM medium (Hyclone), respectively, supplemented with 10% fetal bovine serum (Gibco) and 100 U/mL penicilli/streptomycin (Gibco). For cell treatments, nanoparticles were spun down and then re-dissolved in PBS. The control cells received PBS only.

Hepa 1–6 cells were treated with 20 nM CdCl_2_, 5 nM, 10 nM and 20 nM QDs for 48 hrs, and then cells were washed with PBS and collected in lysis buffer (Promega). The supernatants from cell lysates after centrifugation were used in assays of GSH-Px and MDA. For the assessment of morphological changes upon QD treatment, Hepa 1–6 and J774A.1 cells were treated with 20 nM CdCl_2_, 5 nM, 10 nM and 20 nM QDs for 24 hrs and 48 hrs, and then cells were visualized by phase-contrast microscopy after wash with PBS.

### Flow cytometry

The levels of intracellular ROS were assessed by FACS analysis as previously described [27]. Briefly, cells were incubated with 5 µM of DCF (Molecular Probes; Invitrogen) in medium for 30 mins, and then treated with CdCl_2_ or QDs for 6 hrs. Cells were then collected for flow cytometry analysis after wash with PBS.

### Analyses of MDA level and GSH-Px activity

The MDA level and the GSH-Px activity in the livers and cells were assessed according to the manufacturer's instructions (Wuhan Xinqidi Biological Technology Co., LTD, China). Briefly, supernatants from tissues or cells were added into pre-coated GSH-Px or MDA monoclonal antibody microelisa wells followed by the conventional procedure as described previously [37].

### Statistical analysis

One-way analysis of variance (ANOVA) was used to analyze the mean differences among groups compared to the vehicle control. The difference between two groups was assessed using the independent t-test. P<0.05 was considered statistically significant.
